# Human Single-Cell RNA-Sequencing Data Supports the Hypothesis of X Chromosome Insensitivity but Is Ineffective in Testing the Dosage Compensation Model

**DOI:** 10.1093/molbev/msaf004

**Published:** 2025-02-11

**Authors:** Jiabi Chen, Xiaoshu Chen

**Affiliations:** Department of Immunology and Microbiology, Zhongshan School of Medicine, Sun Yat-sen University, Guangzhou, China; Key Laboratory of Tropical Disease Control, Ministry of Education, Sun Yat-sen University, Guangzhou, China; Department of Immunology and Microbiology, Zhongshan School of Medicine, Sun Yat-sen University, Guangzhou, China; Key Laboratory of Tropical Disease Control, Ministry of Education, Sun Yat-sen University, Guangzhou, China

**Keywords:** dosage compensation, single-cell RNA-sequencing, dosage-insensitive genes

## Abstract

A controversy in evolutionary genetics is whether active dosage compensation is necessary to resolve the gene dosage imbalance between the X chromosome and autosomes. ScRNA-seq data could provide insight into this issue. However, it's crucial to carefully evaluate whether inherent characteristics of scRNA-seq, such as the sparsity of detected genes, might bias the X:AA expression ratio in mammals. This study evaluated two common strategies for selecting genes in the calculation of X:AA, namely, filter-by-expression and filter-by-fraction, with simulated scRNA-seq and bulk RNA-seq datasets. We found that both strategies produce an inflated X:AA, thus artifactually supporting dosage compensation. Analyzing empirical human Smart-seq2 data, results from the filter-by-expression strategy suggested that X-linked genes were more highly expressed than autosomal genes, a pattern that is neither predicted by dosage compensation nor explained by genes escaping X chromosome inactivation. However, the results of the filter-by-fraction strategy are consistent with the simulation. Furthermore, despite biasing for mean expression levels, we found that scRNA-seq data could be used to detect X-to-autosome expression noise differences as small as 10%, which enabled investigation into the distribution of genes that are more likely insensitive to gene dosage changes. Analysis of the empirical Smart-seq2 data revealed a 10% to 15% increase in expression noise for X chromosomes compared with autosomes and a significant depletion of dosage-sensitive genes on X chromosomes. Overall, these results highlight the need to be cautious when interpreting scRNA-seq data, particularly when comparing the expression of different genes, and provide additional evidence for the hypothesis of X chromosome insensitivity.

## Introduction

Due to the degeneration of the mammalian Y chromosome, a dosage imbalance in gene copy number occurs between the X chromosome and the autosomes (Naurin et al. 2010). The solution to this dosage imbalance problem has always been a key element in the evolution of sex chromosomes. In 1967, Susumu Ohno hypothesized that the expression of X-linked genes must be doubled to achieve a gene dosage balance between X-linked and autosomal genes (Ohno 1967). Despite serving as the cornerstone of various evolutionary models of sex chromosome dosage compensation, it was not until 2006 that researchers conducted the first genome-wide test of Ohno's hypothesis using microarray expression profiling (Nguyen and Disteche 2006). The analysis showed that X-linked genes and autosomal genes were expressed at similar levels, supporting Ohno's hypothesis. However, subsequent study revealed that the gene expression detected by microarray has a compression problem, which seriously limits its ability to distinguish whether the expression of X-linked genes is unchanged or doubled (Xiong et al. 2010). With the development of high-throughput experiments, researchers began to study the mRNA levels and protein abundance of X-linked genes using RNA sequencing (RNA-seq) and proteomic data. However, the results of these studies are divided, with some supporting Ohno's hypothesis (Deng et al. 2011; Kharchenko et al. 2011; Lin et al. 2011), while others challenging its applicability to mammals (Xiong et al. 2010; He et al. 2011; Chen and Zhang 2016b; Chen et al. 2020).

**Fig. 1.**
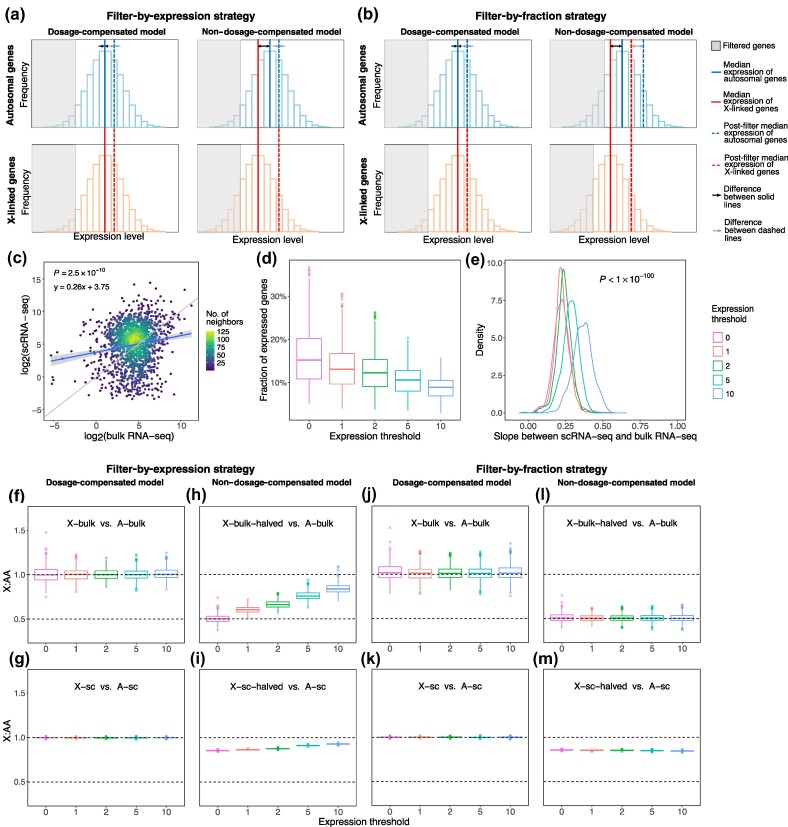
ScRNA-seq preference and its impact on the X:AA ratio. a and b) Schematic diagram of the a) filter-by-expression strategy and b) filter-by-fraction strategy. The blue solid lines represent the median expression of autosomal genes and the red solid lines represent the median expression of X-linked genes for the two dosage models. The gray areas indicate genes filtered based on expression or fraction strategies. The blue and red dashed lines represent the post-filter median expression of genes on autosomes and X chromosomes, respectively. Specifically, in the nondosage-compensated model, filter-by-expression strategy compresses the difference between the blue and red dashed lines (highlighted by gray arrows), which does not reflect the difference between the blue and red solid lines (highlighted by black arrows), while filter-by-fraction strategy prevents such errors. c) Comparison of gene expression levels derived from scRNA-seq and bulk RNA-seq data in human lung samples. The *P*-value indicates the significance of correlation between scRNA-seq and bulk RNA-seq. The diagonal (*x* = *y*) is shown in gray, and the regressed linear model is shown in blue. The gray area indicates the confidence intervals of the slopes. Dots represent individual genes. d) Fractions of genes that are expressed at different expression thresholds in the human lung scRNA-seq data. Dots represent individual cells. e) Distributions of regressed slopes between scRNA-seq and bulk RNA-seq at different expression thresholds in the human lung samples. The *P*-value indicates the significance of slopes <1 by Wilcoxon test, with different thresholds being tested separately and only the maximum *P*-value being displayed. f to i) Simulated X:AA ratios in the dosage-compensated model of bulk RNA-seq f) or scRNA-seq g) and the nondosage-compensated model of bulk RNA-seq h) or scRNA-seq i) via a filter-by-expression strategy. j to m) Simulated X:AA ratios in the dosage-compensated model of bulk RNA-seq j) or scRNA-seq k) and the nondosage-compensated model of bulk RNA-seq l) or scRNA-seq m) via a filter-by-fraction strategy. Simulations were conducted 1,000 times, with each dot representing a simulation. Different expression thresholds are indicated by different colors. The bottom and top of each box are the first and third quartiles, respectively, and the thick line inside the box shows the median. The whiskers extend to the most extreme data point that is no more than 1.5 times the interquartile range from the box edges. Dots show outliers, which lie outside the range shown by the whiskers.

A major methodological distinction leading to the varied conclusions is the filtering method for data points (i.e. genes). The studies supporting Ohno's hypothesis identified expressed genes as those above a specific expression threshold (referred to herein as filter-by-expression, Fig. 1a). Comparisons of the expression of X-linked and autosomal genes were then performed for all expressed genes (Deng et al. 2011; Kharchenko et al. 2011; Lin et al. 2011). However, this approach has been shown to be biased toward the conclusion of increased expression of X-linked genes (Chen et al. 2020). This bias occurs because if the expression level of X-linked genes is not doubled and is therefore lower than that of autosomal genes, the same expression threshold will filter more lowly expressed X-linked genes than autosomal genes, resulting in an artificially overestimated expression level of X-linked genes. Moreover, this bias is exacerbated by increasing the expression threshold, resulting in a greater ratio of X-linked to autosomal gene expression (Chen et al. 2020). Conversely, when comparing a fraction of highly expressed X-linked genes and the same fraction of autosomal genes (referred to herein as filter-by-fraction, Fig. 1b), unbiased estimates can be obtained on whether the expression of X-linked genes is doubled, and the expression ratio of X-linked genes to autosomal genes does not increase with the threshold (Chen et al. 2020). Notably, these different filtering methods have continued to be used in the more recent reassessment of Ohno's hypothesis at the level of single-cell transcriptomes with divided results. These applications of the filter-by-expression or filter-by-fraction methods remain untested for their ability to analyze sparse single-cell transcriptomes (Kulkarni et al. 2019).

Furthermore, following the hypothesis of doubled expression of X-linked genes, Ohno subsequently proposed that one of the X chromosomes in females may be inactivated to maintain a balanced expression of X-linked and autosomal genes (Ohno 1967). The inactivation of one X chromosome in females (referred to as Xi) has been confirmed in many species (Galupa and Heard 2018), which has been considered important empirical evidence supporting Ohno's hypothesis. However, it has also been found that some X-linked genes escape inactivation (referred to as Xe) (Balaton et al. 2018), which disrupts the X-to-autosome expression balance and thus should not be favored or maintained by evolution according to Ohno’s hypothesis. In contrast, a study revealed that escaped genes experienced stronger purifying selection than inactivated genes at both the sequence and gene expression levels (Park et al. 2010).

In an effort to reconcile these empirical observations that conflict with Ohno’s theory, we proposed “X chromosome insensitivity” as an alternative explanation for why X chromosome dosage compensation is not required in some species (Pessia et al. 2012, 2014; Yang and Chen 2019; Chen et al. 2020). An important premise of Ohno's hypothesis is that X-linked genes are dosage-sensitive (Ohno 1967). It can be argued that if the X chromosome had evolved from a nondosage-sensitive proto-autosomal chromosome, then the X-linked genes would not need to be doubled in expression to compensate for dosage halving (Chen et al. 2020). We previously used housekeeping genes as a proxy for dosage-sensitive genes and observed that the X chromosome contained fewer housekeeping genes, thereby supporting our “X chromosome insensitivity” hypothesis (Chen et al. 2020). However, additional stronger evidence is needed for the identification of dosage-sensitive genes.

To address these urgent questions regarding dosage compensation for the mammalian X chromosome, we thoroughly analyzed a variety of high-quality single-cell RNA sequencing (scRNA-seq) datasets from human tissues and reached three major conclusions. First, gene expression data collected by scRNA-seq suffer from sparsity and compression, which causes both existing gene-filtering methods to overestimate X-linked gene expression levels. Second, despite these concerns, scRNA-seq can capture expression noise differences among genes without bias, allowing the estimation of relative dosage sensitivity with reasonable accuracy. Third, the X chromosome was shown to contain more dosage-insensitive genes and be noisier than autosomes. Overall, these results derived from single-cell transcriptomes provide direct support for the X chromosome insensitivity hypothesis, thereby clarifying an important aspect of sex chromosome evolution.

## Results

### Limitations of scRNA-seq Cause Biased Estimation of X:AA

We investigated whether single-cell transcriptomes derived from scRNA-seq are suitable for direct estimation of the X-to-autosome expression ratio (X:AA). To this end, a Smart-seq2 dataset containing 23 human tissues (The Tabula Sapiens Consortium 2022) was compared with bulk RNA-seq data (Fig. 1c and supplementary fig. S1 and table S1, Supplementary Material online). On the one hand, the expression levels obtained by the two methods were significantly similar, suggesting that scRNA-seq can generally recapitulate bulk RNA-seq. On the other hand, scRNA-seq data are limited (Kharchenko et al. 2014; Bouland et al. 2023) by gene expression sparsity and compression. More specifically, the sparsity of scRNA-seq data is evident, as only approximately 15% of genes were detected per cell (Fig. 1d and supplementary fig. S2, Supplementary Material online). Lowly expressed genes are extremely difficult to detect by scRNA-seq, effectively increasing the threshold for expression detection and reducing the fraction of expressed genes. In addition, a linear regression between the bulk RNA-seq data and the scRNA-seq data yielded a slope less than one and an intercept moderately greater than zero (Fig. 1c and e and supplementary fig. S3, Supplementary Material online), indicating that highly expressed genes in the bulk RNA-seq data were underestimated in the scRNA-seq data, whereas genes with low expression were overestimated. We called this phenomenon “compression” of expression in scRNA-seq, as it compresses gene expression differences detected by bulk RNA-seq, thereby biasing the X:AA toward 1, as previously observed in microarray experiments (Xiong et al. 2010).

To verify that the sparsity and compression issues inherent to scRNA-seq-based gene expression levels can indeed bias the X:AA estimation, we performed a computational simulation (Chen et al. 2020). The simulation involved two main steps: (i) the generation of expression data for both dosage-compensation and nondosage-compensation models (supplementary fig. S4, Supplementary Material online) and (ii) the application of different strategies to determine X:AA. For step (i), all autosomal protein-coding genes in the bulk RNA-seq data of a lung sample (supplementary table S1, Supplementary Material online) were selected as A-bulk, and then a subset of these genes was randomly selected as X-bulk. The number of genes chosen for X-bulk was the same as the number of actual human X-linked protein-coding genes. The expression levels of the A-bulk and X-bulk genes were combined to create a dosage-compensated dataset of bulk RNA-seq data. Another dataset, called the nondosage-compensated dataset, was created by combining A-bulk genes with expression-halved X-bulk genes (X-bulk-halved). To generate the corresponding scRNA-seq data for dosage-compensated or nondosage-compensated dataset, genes in A-bulk, X-bulk, and X-bulk-halved were randomly selected based on the distribution of the fraction of expressed genes and the detection probability of each gene in the scRNA-seq data of the human lung sample (The Tabula Sapiens Consortium 2022). They were then transformed into A-sc, X-sc, and X-sc-halved according to the linear model regressed for scRNA-seq on bulk RNA-seq (Fig. 1c; see Materials and Methods). A-sc and X-sc were then merged into a dosage-compensated dataset, and A-sc and X-sc-halved were merged into a nondosage-compensated dataset.

In step (ii), we applied both filter-by-expression and filter-by-fraction strategies to calculate X:AA for the four datasets obtained from step (i). In the filter-by-expression strategy, autosomal and X-linked genes that are expressed above a certain expression threshold are selected for calculating X:AA. This filter, regardless of the threshold values used, produced the expected X:AA = 1 when applied to the dosage-compensated datasets in bulk RNA-seq (Fig. 1f) and in scRNA-seq (Fig. 1g), but overestimated X:AA to different degrees when applied to the nondosage-compensated datasets (Fig. 1h and i). The overestimated X:AA implies some degree of dosage compensation, even if no compensation is present. Notably, overestimation of X:AA in the sc-halved sample (Fig. 1i) was more pronounced than that in the bulk-halved sample (Fig. 1h). These results indicate that the filter-by-expression strategy has an intrinsic bias in favor of dosage compensation, which is further exaggerated in scRNA-seq due to its expression compression effect.

With the filter-by-fraction strategy, the same fraction of the most highly expressed autosomal and X-linked genes is considered, which avoids excessive removal of genes with low expression from the X chromosome (Chen et al. 2020). In both the dosage-compensated datasets of bulk RNA-seq and scRNA-seq, the filter-by-fraction strategy faithfully reproduced the expected X:AA = 1 (Fig. 1j and k). However, in the nondosage-compensated datasets, the expected X:AA = 0.5 was found only in the bulk-halved sample (Fig. 1l), not in the sc-halved sample (Fig. 1m; Wilcoxon test: X:AA > 0.5, *P* < 1 × 10^−100^; X:AA < 1, *P* < 1 × 10^−100^). These results suggest that X:AA based on the filter-by-fraction strategy is unbiased in bulk RNA-seq data but not in scRNA-seq data due to the expression compression effect. Overall, we conclude that scRNA-seq provides a biased, overestimated X:AA in favor of dosage compensation of X-linked genes.

### The X:AA Ratios of scRNA-seq in Human Tissues

**Fig. 2.**
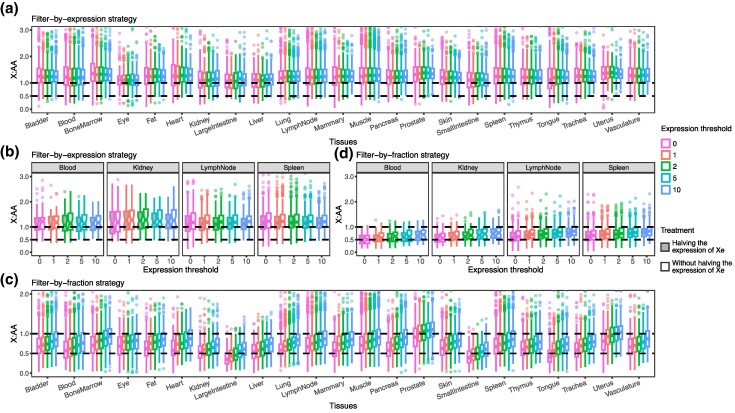
X:AA ratios from scRNA-seq data in human tissues. a) X:AA ratios calculated by the filter-by-expression strategy. b) X:AA ratios of B lymphocytes calculated by the filter-by-expression strategy with and without halving the expression of Xe. c) X:AA ratios calculated by the filter-by-fraction strategy. d) X:AA ratios of B lymphocytes calculated by the filter-by-fraction strategy with and without halving the expression of Xe. Dots represent individual cells. Different expression thresholds are indicated by different colors. The bottom and top of each box are the first and third quartiles, respectively, and the band inside the box shows the median. The whiskers extend to the most extreme data point that is no more than 1.5 times the interquartile range from the box edges. Dots show outliers, which lie outside the range shown by the whiskers.

Based on the above simulation results, we examined empirical X:AA using scRNA-seq datasets from 23 human tissues (The Tabula Sapiens Consortium 2022). We first applied the filter-by-expression strategy. Interestingly, the median X:AA using this strategy was >1 (Fig. 2a; Wilcoxon test: *P* < 1 × 10^−6^), an observation that was stable regardless of the expression thresholds used. This value of X:AA cannot be explained by the dosage-compensation model or the nondosage-compensation model, according to the simulation results. We speculate that the sparsity of the scRNA-seq data could have contributed to this pattern. In particular, scRNA-seq preferentially captured genes with high expression, especially those Xe genes escaping X chromosome inactivation (XCI), the expression of which is elevated due to dosage compensation (as hypothesized by Ohno) and doubled gene dosage (both copies are active). To test this hypothesis, 68 Xe genes (out of 508 X-linked genes) were retrieved from a study that investigated X-linked allele imbalance in lymphoblastic cell lines of females with skewed XCI (Cotton et al. 2013). The X:AA ratios of B lymphocytes in the four tissues were calculated before and after Xe expression was halved (Fig. 2b). Despite a significant decrease in X:AA (Wilcoxon signed-rank test: *P* < 1 × 10^−53^) after Xe expression was halved, the median X:AA ratios for these four types of tissues were still >1 (Wilcoxon test: *P* < 1 × 10^−27^). A similar conclusion was reached using the Xe genes obtained from another study (Carrel and Willard 2005) (supplementary fig. S5, Supplementary Material online). Therefore, the observed X:AA > 1 cannot be fully explained by Xe. These results suggest that the filter-by-expression approach is not appropriate for calculating the X:AA ratio using scRNA-seq data.

Next, we calculated X:AA for the same scRNA-seq dataset using the filter-by-fraction strategy. Most tissues had a median X:AA ratio between 0.5 and 1 (Fig. 2c; Wilcoxon test: X:AA > 0.5, *P* < 1 × 10^−3^; X:AA < 1, *P* < 1 × 10^−5^), which is consistent with our simulation of the nondosage-compensation model. That is, the X:AA ratio is modestly >0.5 due to the expression compression effect of scRNA-seq but rarely reaches the expected dosage-compensation value of 1. As the filter-by-fraction strategy distinguishes the two alternative models better than the filter-by-expression strategy, the results are clearly compatible with nondosage compensation rather than dosage compensation. Notably, when we increased the expression filtering thresholds, the X:AA ratio increased slightly, whereas the simulated X:AA ratio did not change. This pattern cannot be explained by genes escaping XCI, as even when the expression levels of Xe were halved, the increase of X:AA with filtering thresholds persisted (Fig. 2d), suggesting that other unknown factors may be involved.

Ohno’s hypothesis also predicts that the expression levels of X-linked genes are doubled during the early stages of the transformation of the proto-X chromosome into the X chromosome (Ohno 1967). Humans and chickens have different sex determination systems, with humans having the XY system while chickens have the ZW system. Human X-linked genes with orthologs in chickens are found on chicken autosomes and therefore represent early evolutionary stages of the human proto-X chromosome. We extracted human genes with 1-to-1 orthologs in chickens and repeated the above analyses of X:AA. The median X:AA ratios for 1-to-1 orthologs were similar to those found for all protein-coding genes (supplementary fig. S6, Supplementary Material online).

Together, these empirical results suggested that the filter-by-expression strategy is inappropriate and that the results of the filter-by-fraction strategy in scRNA-seq data of human tissues are more consistent with the nondosage-compensation model. However, the X:AA ratios obtained with the filter-by-fraction strategy may still be overestimated due to the expression compression effect of scRNA-seq.

### The Impact of scRNA-seq Characteristics on the Expression Noise of X Chromosomes and Autosomes

To explain the lack of dosage compensation for X-linked genes, we proposed the X chromosome insensitivity hypothesis (Chen et al. 2020), which suggests that the mammalian X chromosome has evolved from an autosome depleted of dosage-sensitive genes. As dosage-sensitive genes should display lower gene expression heterogeneity or expression noise (Yin et al. 2009; Mullon et al. 2015), a key prediction of the X chromosome insensitivity hypothesis is that the expression noise of X-linked genes is greater than that of autosomal genes. Our previous study tested this prediction by identifying housekeeping genes, which presumably have low expression noise, using bulk RNA-seq data (Chen et al. 2020), and by direct estimation of gene expression noise using scRNA-seq data from embryonic stem cells (Yang and Chen 2019). Below, we revisit the scRNA-seq-based estimation of expression noise by examining the impact of expression sparsity and compression effects of scRNA-seq via simulation.

On the basis of a previous approach (Newman et al. 2006; Chen and Zhang 2016a), we were able to estimate the level of expression noise of a gene (hereafter referred to as the focal gene) based on the *D*ifference between its expression variance (measured by the coefficient of variation, or CV) and the *M*edian expression variance of genes with similar expression levels (DM), thereby controlling the effects of expression mean on expression variance (Newman et al. 2006) (supplementary fig. S7, Supplementary Material online; see Materials and Methods). The simulated scRNA-seq datasets of expression noise were constructed as follows (supplementary fig. S8, Supplementary Material online). On the basis of the scRNA-seq data of human female lung samples (The Tabula Sapiens Consortium 2022), each autosomal gene was assigned an expression variance randomly extracted from another autosomal gene with a similar expression level to the focal gene (see Materials and Methods). This dataset of autosomal genes with reassigned expression variances (and therefore DMs) was denoted A-sc′. The second dataset, denoted X-sc′, consisted of a random subset of autosomal genes that contained the same number of genes as X-linked genes. The expression variance of each gene in X-sc′ was increased to various levels (see below) to yield a third dataset named X-sc-noisy′, which was used to assess the statistical power for the designated noise increases. To simulate the nondosage-compensation model, the expression level of X-sc′ was halved, and the expression variance was reassigned from another gene with a similar expression level of the focal gene (i.e. the halved expression), resulting in the X-sc-halved′ dataset. Finally, an X-sc-halved-noisy′ dataset was constructed by increasing the expression variance in the X-sc-halved′ dataset to various levels.

**Fig. 3.**
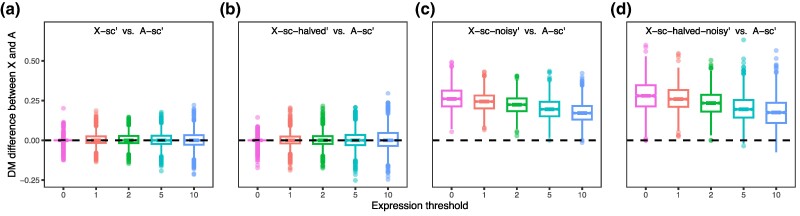
Impact of scRNA-seq on estimating X-to-autosome expression noise differences. The X-to-autosome difference in DM estimated by simulated scRNA-seq datasets with a) the original mean FPKM or CV, b) halving the mean FPKM and maintaining the CV, c) maintaining the mean FPKM and increasing the CV, and d) halving the mean FPKM and increasing the CV. The simulated CVs were chosen at random from 100 genes with the closest expression levels. Simulations were conducted 1,000 times, with each dot representing a simulation. Different expression thresholds are indicated by different colors. The bottom and top of each box are the first and third quartiles, respectively, and the thick line inside the box shows the median. The whiskers extend to the most extreme data point that is no more than 1.5 times the interquartile range from the box edges. Dots show outliers, which lie outside the range shown by the whiskers.

We then applied the filter-by-fraction strategy before comparing the DMs of X-linked and autosomal genes. Two important findings were observed. First, the DM difference between X-sc′ and A-sc′ was 0, regardless of the filtering threshold used, indicating that both datasets had the same level of expression noise (Fig. 3a). Second, the DM difference between X-sc-halved′ and A-sc′ was also 0 (Fig. 3b), indicating that the DM difference can still be estimated without bias even with a twofold change in the expression level. Notably, the variance of DM difference was significantly greater in the comparison involving X-sc-halved′ relative to that involving X-sc′ (Fig. 3b; Levene test: *P* < 1 × 10^−2^), suggesting that the sparsity of scRNA-seq might inflate the DM variance for genes with low expression levels. These observations were also robust to the number of genes with similar expression levels used to extract the simulated variance (supplementary fig. S9, Supplementary Material online).

We next assessed whether an increase in expression noise in X-linked genes could be detected by the DM-based method using the X-sc-noisy′ and X-sc-halved-noisy′ datasets. X-linked genes exhibited significantly greater DM than autosomes when the expression variance increased by 10% (Fig. 3c; Wilcoxon test: *P* < 1 × 10^−100^), suggesting that scRNA-seq can detect chromosome-scale expression noise differences as small as 10%. In particular, the difference in DM was significantly influenced by an increase in variance rather than a halving of expression levels when the two changes occurred simultaneously (Fig. 3b to d). As the expression threshold increased, the difference detected in DM gradually decreased but remained above 0 (Fig. 3c and d). Overall, despite the expression sparsity and compression effects, scRNA-seq can still distinguish chromosome-scale increases in expression noise. It is therefore appropriate to assess for gene expression noise using scRNA-seq-based DM values.

### The Expression Noise of X-linked and Autosomal Genes Across Human Tissues

**Fig. 4.**
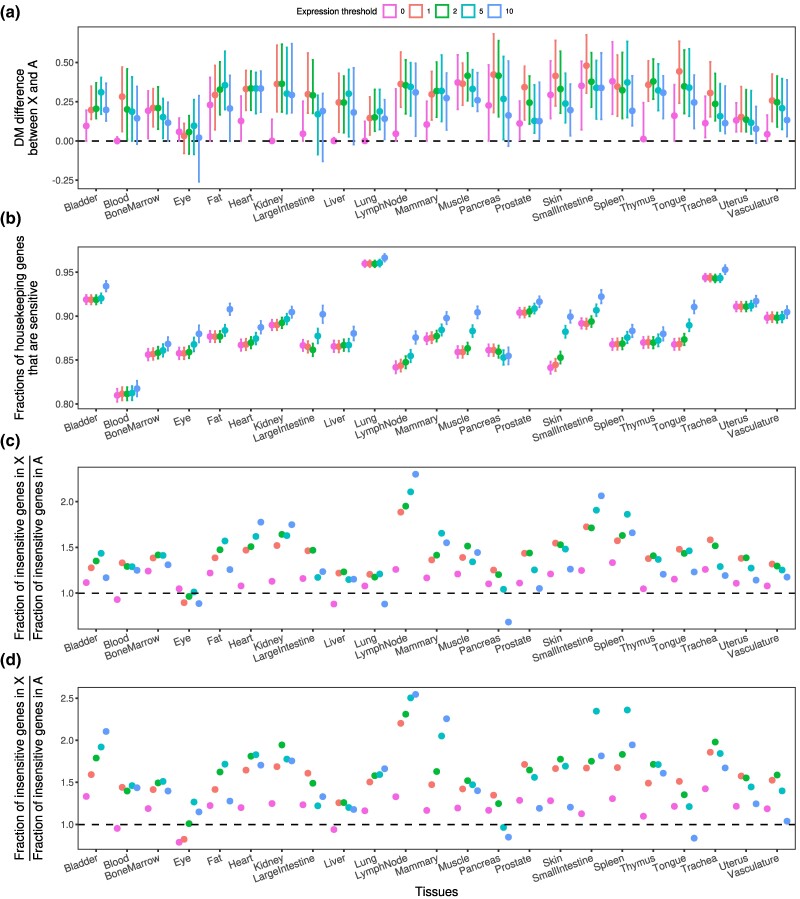
X-to-autosome expression noise differences estimated by scRNA-seq in human tissues. a) Differences in DM between X-linked genes and autosomal genes in human tissues. Dots represent the actual DM differences, and error bars represent a CI of 90% according to bootstrapping. b) Fractions of housekeeping genes that are classified as dosage sensitive by DM. Dots represent the actual fractions, and error bars represent SE. c and d) The ratio between the fraction of insensitive genes found in X-linked genes and autosomal genes with c) all protein-coding genes and d) 1-to-1 orthologs in chickens. Different expression thresholds are indicated by different colors.

Following the validation of our hypothesis using scRNA-seq data, we investigated whether X-linked genes are generally less sensitive to expression noise than autosomal genes using the scRNA-seq dataset from 23 human tissues (The Tabula Sapiens Consortium 2022). Almost all tissues showed a greater median DM on the X chromosome regardless of the threshold (Fig. 4a), suggesting greater expression noise and therefore lower dosage sensitivity for the X chromosome. The X-to-autosome difference in DM was approximately 0.25, indicating that the expression variance of the X chromosome was approximately 10% to 15% greater than that of autosomes (supplementary fig. S10, Supplementary Material online).

To further test our hypothesis, we classified genes as insensitive (or sensitive) to dosage differences according to whether the gene exhibited a DM > 1 (or ≤1). The biological relevance of this criterion is demonstrated by the finding that more than 85% of housekeeping genes (see Materials and Methods), which are widely recognized to be dosage sensitive and to have extremely low expression noise (Bar-Even et al. 2006), were categorized as sensitive genes (Fig. 4b). In most tissues, the fraction of insensitive genes on X chromosome was greater than that on autosomes, regardless of the expression filtering thresholds, approximately 1.5 times higher (Fig. 4c). A similar pattern was recapitulated using genes with 1-to-1 orthologs in chickens (Fig. 4d), which indicates that the proto-X-linked genes have displayed a higher degree of insensitivity than the proto-autosomal genes, supporting that the proto-X chromosome originated from an insensitive rather than a sensitive chromosome.

Collectively, our analyses of dosage-sensitive genes using scRNA-seq-based expression noise support the X chromosome insensitivity hypothesis. Because the X chromosome evolved from a generally insensitive proto-autosome, dosage compensation, let alone chromosome-wide doubled expression, is unnecessary for most X-linked genes.

## Discussion

In this study, we identified the expression sparsity and compression effects of scRNA-seq, a phenomenon that severely impedes the estimation of the X:AA ratio. The results of subsequent computational simulations based on a nondosage-compensation model indicate that the expression compression effects will result in an X:AA ratio above 0.75, creating an artificial pattern of dosage compensation. This false support for dosage compensation occurs regardless of whether the filter-by-expression or filter-by-fraction methods are used to select genes for analysis, with any specific filtering thresholds. Furthermore, the empirical scRNA-seq data from 23 human tissues (The Tabula Sapiens Consortium 2022) showed an increase in the ratio of X:AA, which was generally in agreement with the simulation results. As another test of dosage compensation that is not influenced by the expression compression effect of scRNA-seq, we approximated the dosage sensitivity of genes based on their expression noise. After demonstrating through simulation that as little as a 10% increase in expression variance at the chromosome level can be detected by scRNA-seq, we found that X-linked genes exhibited greater expression noise than autosomal genes, and X chromosome had more insensitive genes. In summary, these results support the hypothesis that most X-linked genes are likely dosage insensitive, indicating that they do not require an active mechanism of dosage compensation.

It is worth emphasizing again here that the filter-by-expression strategy, when applied to bulk RNA-seq data, produces a bias in the estimation of the X chromosome-to-autosome expression ratio in favor of X:AA = 1, which becomes more pronounced as the expression threshold increases. In contrast, the filter-by-fraction strategy provides an accurate estimation of X:AA = 1 under a dosage-compensation model and X:AA = 0.5 under a nondosage-compensation model. However, for scRNA-seq data, the X:AA ratios increased to a level between 0.5 and 1, indicating that the filter-by-fraction strategy is also influenced by the expression compression effects of scRNA-seq data. Furthermore, these results raise concerns about underestimated expression differences among genes when using scRNA-seq data. To interpret scRNA-seq data more effectively, it may be useful to develop an algorithm that estimates expression differences or ratios among genes without bias.

Surprisingly, the X:AA ratios in 23 human tissues estimated by empirical scRNA-seq data and the filter-by-expression strategy exceeded 1. This result cannot be explained by either dosage compensation or genes escaping XCI. We suspected that this result was due to insufficient identification of Xe or, alternatively, to the sparsity of the scRNA-seq data. Despite attempting to identify Xe via scRNA-seq, we were unsuccessful due to insufficient single-nucleotide polymorphism loci in the existing data for human cell lines and high sequencing error rates in scRNA-seq data.

To evaluate Ohno’s hypothesis and its alternatives while circumventing the expression sparsity and compression effects of scRNA-seq, we estimated gene expression noise, or DM, which allows us to identify dosage-sensitive or -insensitive genes. The significant overlap between housekeeping genes and dosage-sensitive genes, as well as evidence from computational simulations, suggested that scRNA-seq-based DM can indeed facilitate estimation of dosage sensitivity. In other words, gene expression noise, rather than expression mean, may be a more useful scRNA-seq-based measurement to address the problem of dosage compensation.

The hypothesis of X chromosome insensitivity proposes that the proto-X chromosome originated from an insensitive autosome, explaining why X-linked genes do not require double expression in mammals despite the imbalance in dosage between autosomes and the X chromosome (Chen et al. 2020). Bulk RNA-seq data has previously demonstrated that there are very few housekeeping genes on the proto-X chromosome (Chen et al. 2020), whereas scRNA-seq data here reveal a relatively high fraction of noisy genes on the X chromosome for both protein-coding genes and genes with 1-to-1 orthologs in chickens, further supporting this hypothesis. Nevertheless, to understand why mammals chose insensitive ancestral autosomes as the proto-X chromosome, and what selective pressure preserves the insensitivity of the X chromosome, additional explanations from other hypotheses are necessary. The “Faster X” hypothesis (Mank et al. 2007), for example, may suggest that the rapid evolution of X chromosome makes it a less desirable location for the dosage-sensitive housekeeping genes.

To increase the generality of our conclusions, we also used 10× Genomics scRNA-seq data since they covered a broader range of species and tissues (Tabula Muris Consortium 2018; Raredon et al. 2019; Li et al. 2022). However, the 10× Genomics scRNA-seq data were insufficient to determine dosage compensation on the X chromosome (supplementary fig. S11, Supplementary Material online), largely due to their low gene expression resolution and severe gene sparsity. Smart-seq2 has a higher resolution and milder sparsity issue than 10× Genomics, but it presents another challenge in terms of the limited data available for the analysis of multiple species and tissues. Until now, datasets of this type have only been published for humans (The Tabula Sapiens Consortium 2022), mice (Tabula Muris Consortium 2018), and flies (Li et al. 2022). Since contrasting conclusions have been reported for mouse data (Kimmel et al. 2019), implicating quality issues therein, we only further examined the Smart-seq2 dataset of flies. Unlike mammals, flies possess dosage compensation on the X chromosome. Male flies double the expression of X-linked genes through testis-specific lethal complexes, compensating for their halved doses compared with autosomal genes (Nozawa et al. 2014). According to our analysis, the filter-by-fraction strategy is capable of identifying the same expression levels of X-linked genes and autosomal genes with greater accuracy than the filter-by-expression strategy (supplementary fig. S12a and b, Supplementary Material online). Moreover, we found that the expression noise of X chromosome in flies was lower than that of autosomes, which is consistent with the results of housekeeping genes (Chen et al. 2020), although the difference is only 5% (supplementary fig. S12c to k, Supplementary Material online). The above findings support our hypothesis that, if the X chromosome originates from a sensitive autosome, a mechanism is required to facilitate upregulation of the entire X chromosome, and vice versa (Chen et al. 2020). Nevertheless, the hypothesis of X chromosome insensitivity shall be further examined with high-quality scRNA-seq data in the future, by quantifying and comparing the sensitivity of sex chromosomes across species, and by estimating the sensitivity of ancestral chromosomes.

## Materials and Methods

### Transcriptomic Data Collection and Processing

Bulk RNA-seq datasets were selected from the Gene Expression Omnibus (Clough et al. 2024) with the following criteria: (i) The tissue and sex of the sample matched those used for human scRNA-seq (The Tabula Sapiens Consortium 2022). (ii) Bulk RNA-seq data were not derived from pathological, stress-treated, or genetically modified samples. (iii) If there were multiple data, the one with the greatest sequencing depth was chosen. The final bulk RNA-seq datasets consisted of 25 SRA files from 22 tissues (supplementary table S1, Supplementary Material online). We then downloaded the SRA files using the SRA Toolkit (https://github.com/ncbi/sra-tools), removed adapters and low-quality reads with fastp (Chen et al. 2018), mapped all the clean reads to the human genome with HISAT2 (Kim et al. 2019), and quantified expression levels with StringTie (Pertea et al. 2015) to obtain fragments per kilobase of transcript per million mapped reads (FPKM) matrices that were corrected for both gene length and sequencing depth (Mortazavi et al. 2008).

The scRNA-seq datasets based on the Smart-seq2 protocol were obtained from the Tabula Sapiens Consortium (The Tabula Sapiens Consortium 2022) and Fly Cell Atlas (Li et al. 2022). Those cells expressing <1,500 unique genes and tissues containing <50 cells were considered low-quality and were excluded from further analysis (supplementary table S2, Supplementary Material online). The raw count matrices were then transformed into FPKM matrices. The scRNA-seq datasets based on the 10× Genomics protocol were obtained from Mammalian Lungs (Raredon et al. 2019), Tabula Muris (Tabula Muris Consortium 2018), and Fly Cell Atlas (Li et al. 2022). Cells with fewer than 500 unique genes detected were considered low quality and were excluded from further analysis (supplementary table S3, Supplementary Material online). The raw count matrices were directly used without transformation.

The human genome sequence, along with annotations of all protein-coding genes and homology (1-to-1 orthologs) with chicken genes, was downloaded from Ensembl version 89 (www.ensembl.org). The annotations of mouse and fly were downloaded from GENCODE release M10 (https://www.gencodegenes.org/) and Ensembl version 109, respectively. The analysis was limited to protein-coding genes, unless otherwise indicated. Several possible cases were tested using 1-to-1 orthologs of protein-coding genes related to humans and chickens.

### Comparison of scRNA-seq and Bulk RNA-seq Expression Levels

In each cell, the logarithmic expression levels of protein-coding genes derived from scRNA-seq and those derived from bulk RNA-seq were analyzed using Pearson correlation analysis and linear model fitting. The Wilcoxon test was used to determine whether the regression slopes obtained from the linear model were <1. The above analysis was repeated with different expression thresholds (FPKM >0, 1, 2, 5, and 10).

### Simulating Transcriptome Data for Comparison of Expression Levels

Based on a bulk RNA-seq dataset of the female lung sample (accession number SRR2102538, supplementary table S1, Supplementary Material online), we selected all autosomal protein-coding genes as A-bulk (supplementary fig. S4, Supplementary Material online). A subset of A-bulk containing the same number of human X-linked protein-coding genes was chosen at random as X-bulk, and its expression level was further halved as X-bulk-halved. Genes of the A-bulk, X-bulk, and X-bulk-halved were selected randomly based on the following criteria: First, the number of genes should correspond to the distribution of fractions of expressed genes in the lung sample from the Tabula Sapiens Consortium (The Tabula Sapiens Consortium 2022) (supplementary table S2, Supplementary Material online). Second, a gene’s probability of being selected should follow its detection probability in this sample. They were then transformed into A-sc, X-sc, and X-sc-halved, respectively, based on the median regression slope between the bulk RNA-seq lung sample and the scRNA-seq lung sample, with the following formula: FPKM_scRNA-seq_ = 2 ^ (0.26 × log_2_(FPKM_bulk_  _RNA-seq_) + 3.75). In each A-bulk, X-bulk, and X-bulk-halved, 100 A-sc, X-sc, and X-sc-halved were sampled, representing 100 individual cells.

For bulk RNA-seq, we repeated the procedure 1,000 times to build 1,000 dosage-compensated datasets, each containing one A-bulk and one X-bulk, and 1,000 nondosage-compensated datasets, each containing one A-bulk and one X-bulk halved. Similarly, for scRNA-seq, 1,000 dosage-compensated datasets were constructed, each containing 100 A-sc and 100 X-sc, as well as 1,000 nondosage-compensated datasets, each containing 100 A-sc and 100 X-sc-halved.

### Calculation of the Expression Ratio of X-Linked Genes to Autosomal Genes

For the filter-by-expression strategy, we first extracted the expressed X-linked and autosomal genes in each transcriptional profile (bulk or scRNA-seq) with an FPKM >0, 1, 2, 5, or 10. At each expression threshold, the ratio of the median expression of the extracted X-linked genes to the median expression of the extracted autosomal genes was then calculated to determine the X:AA ratio.

For the filter-by-fraction strategy, we first calculated the expression fractions of X-linked genes and autosomal genes separately in each transcriptional profile with an FPKM >0, 1, 2, 5, or 10. At each expression threshold, the lower of the X-linked expression fraction or the autosomal expression fraction was selected. This selected expression fraction was used to extract the most highly expressed X-linked genes and autosomal genes separately. The X:AA ratio was then calculated by dividing the median expression of the extracted X-linked genes by the median expression of the extracted autosomal genes.

A total of 68 Xe genes were identified in a study that examined 508 X-linked genes in most lymphoblastic cells of women with skewed XCI (Cotton et al. 2013). In our analyzed scRNA-seq dataset, there were four types of tissue, including blood, kidney, lymph node, and spleen, with B lymphocyte cells (supplementary table S4, Supplementary Material online). In another study, Xe genes were identified among 612 X-linked genes in 9 rodent/human fibroblast hybrids (Carrel and Willard 2005). According to this study, we extracted 55 protein-coding genes that had escaped inactivation in at least 7 hybrids as Xe genes. The scRNA-seq dataset contains fibroblast cells found in the bladder, lung, mammary, thymus, uterus, and vasculature (supplementary table S4, Supplementary Material online). Pseudoautosomal genes have already been identified as Xe genes in these two studies (Carrel and Willard 2005; Cotton et al. 2013). In the corresponding tissues, we halved the expression of the Xe genes and calculated the X:AA ratio as described above.

### Estimation of Expression Noise

For each tissue of the scRNA-seq dataset, we selected the donor with the largest number of cells for downstream analysis to reduce the impact of among-donor differences on expression noise (supplementary table S2, Supplementary Material online). For each gene, we calculated the mean and CV of the FPKM in all cells. As expression variance and expression mean are positively correlated (Newman et al. 2006; Chen and Zhang 2016a), we used a previously established approach to correct for the impact of expression mean on expression variance in order to compare expression noise among genes more accurately (Newman et al. 2006). In particular, genes were first sorted according to their expression level. Next, the expected CV of the focal gene was determined by the median CV of the 100 genes whose expression levels are closest to the focal gene, 50 of which had a lower expression level and 50 of which had a higher expression level. The difference between the focal CV and the expected CV (DM) was used as a measurement of expression noise (supplementary fig. S7 and supplementary tables S5 and S6, Supplementary Material online) (Newman et al. 2006; Chen and Zhang 2016a). Moreover, this calculation was also performed with 50 genes instead of 100 as the range of genes with the closest expression level.

### Simulating Single-Cell Transcriptomes for Expression Noise Comparison

Using the Smart-seq2 data of female lung samples from The Tabula Sapiens Consortium (2022), we calculated the mean and CV of the FPKM for each autosomal protein-coding gene. These mean FPKMs were used as the mean FPKMs in the simulation A-sc′ dataset (supplementary fig. S8, Supplementary Material online). The CV for a focal gene in A-sc′ was selected at random from the CVs of 100 genes with the closest mean FPKMs to the focal gene, 50 of which had a lower mean FPKMs and 50 of which had a higher mean FPKMs. For the simulated X-linked genes, the following four treatments were applied. (i) The mean FPKMs of the same number of X-linked protein-coding genes were extracted from A-sc′ and used as the mean FPKMs of the simulated X-sc′ dataset. The CV for a focal gene in X-sc′ was selected from 100 CVs with the closest mean FPKMs as in A-sc′. (ii) The mean FPKMs in X-sc′ were halved, and one CV was selected as the focal gene's CV from 100 CVs with mean FPKMs closest to the halved value. (iii) The mean FPKMs in X-sc′ remained unchanged, while the CVs of focal genes increased by factors of 1.05, 1.1, 1.15, or more. (iv) The second and third treatments were combined by halving the mean FPKM in X-sc′, extracting the CV of the focal gene from 100 CVs with the closest mean FPKMs to the halved value, and then increasing those CVs by factors of 1.05, 1.1, 1.15, or more. In cases where the CV increased beyond the maximum CV found across all genes, the maximum CV was used instead. The CVs were further transformed into DM based on their respective mean FPKMs and expected CVs. This procedure was repeated 1,000 times, resulting in 1,000 A-sc′, X-sc′, X-sc-halved′, X-sc-noisy′, and X-sc-halved-noisy′ datasets. Moreover, this simulation was also repeated with 50 genes instead of 100 as the range of genes when choosing the simulated CV for a focal gene. Additionally, we conducted these simulations on flies using Smart-seq2 data generated from tracheal tissue of the Fly Cell Atlas (Li et al. 2022), with the CVs of focal genes not increasing but rather decreasing to 98%, 96%, and 94%, respectively.

### Comparison of Expression Noise between X-Linked Genes and Autosomal Genes

For both empirical and simulated scRNA-seq data, we first adopted the filter-by-fraction strategy to select X-linked and autosomal genes for comparison at different expression thresholds. Next, we subtracted the median DM of X-linked genes from the median DM of autosomal genes to calculate the DM difference between the X chromosomes and autosomes. The 90% CI for the difference of empirical data was estimated by bootstrapping the X-linked and autosomal genes 1,000 times.

The housekeeping genes were defined as those with a mean FPKM >2 across all tissues in the scRNA-seq data (supplementary table S7, Supplementary Material online). In each tissue, genes with DM > 1 were classified as insensitive genes, while the rest were classified as sensitive genes (supplementary table S8, Supplementary Material online). The fraction of sensitive genes among the housekeeping genes was then calculated. Moreover, we calculated the fraction of insensitive genes on X chromosomes and autosomes, and compared these two fractions. A quotient >1 indicates that the X chromosome tends to be insensitive to dosage changes compared with autosomes.

## Supplementary Material

msaf004_Supplementary_Data

## Data Availability

The data underlying this article are publicly available from various sources, as listed in supplementary tables S1 to S3, Supplementary Material online.
