# Supplementary figures and images for: Human Single-Cell RNA-Sequencing Data Supports the Hypothesis of X Chromosome Insensitivity but Is Ineffective in Testing the Dosage Compensation Model
Source: Mol Biol Evol. 2025 Feb 11;42(2):msaf004. doi: 10.1093/molbev/msaf004 (PMC11811734; doi:10.1093/molbev/msaf004)

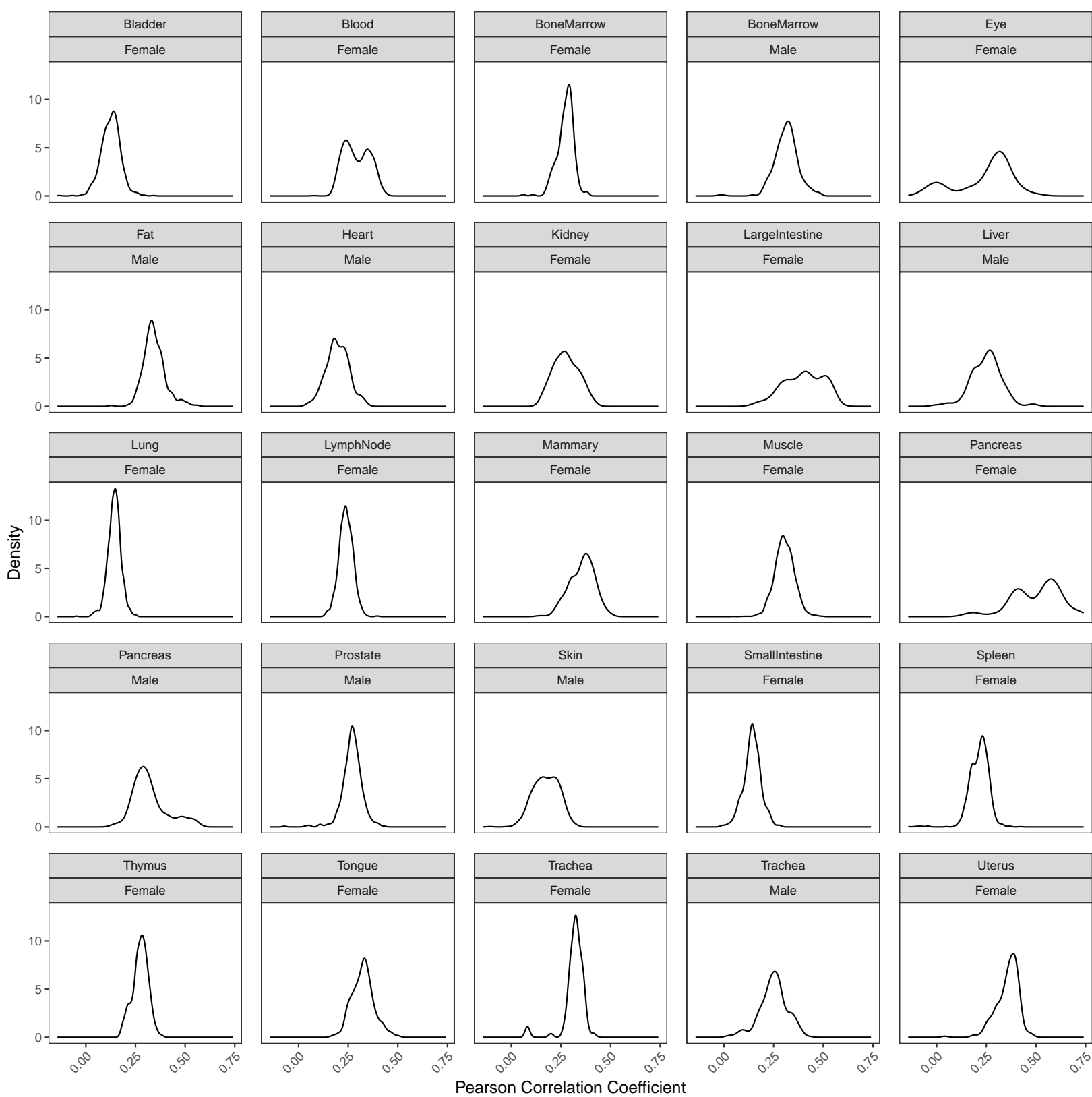

**Fig. S1**

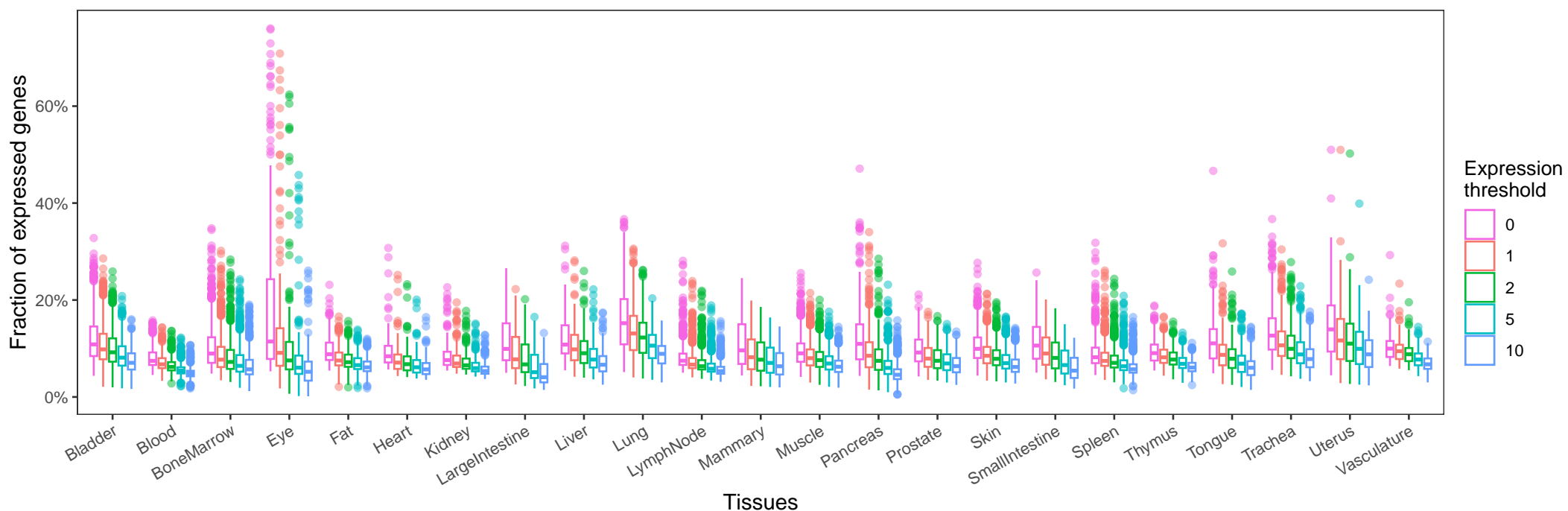

**Fig. S2**

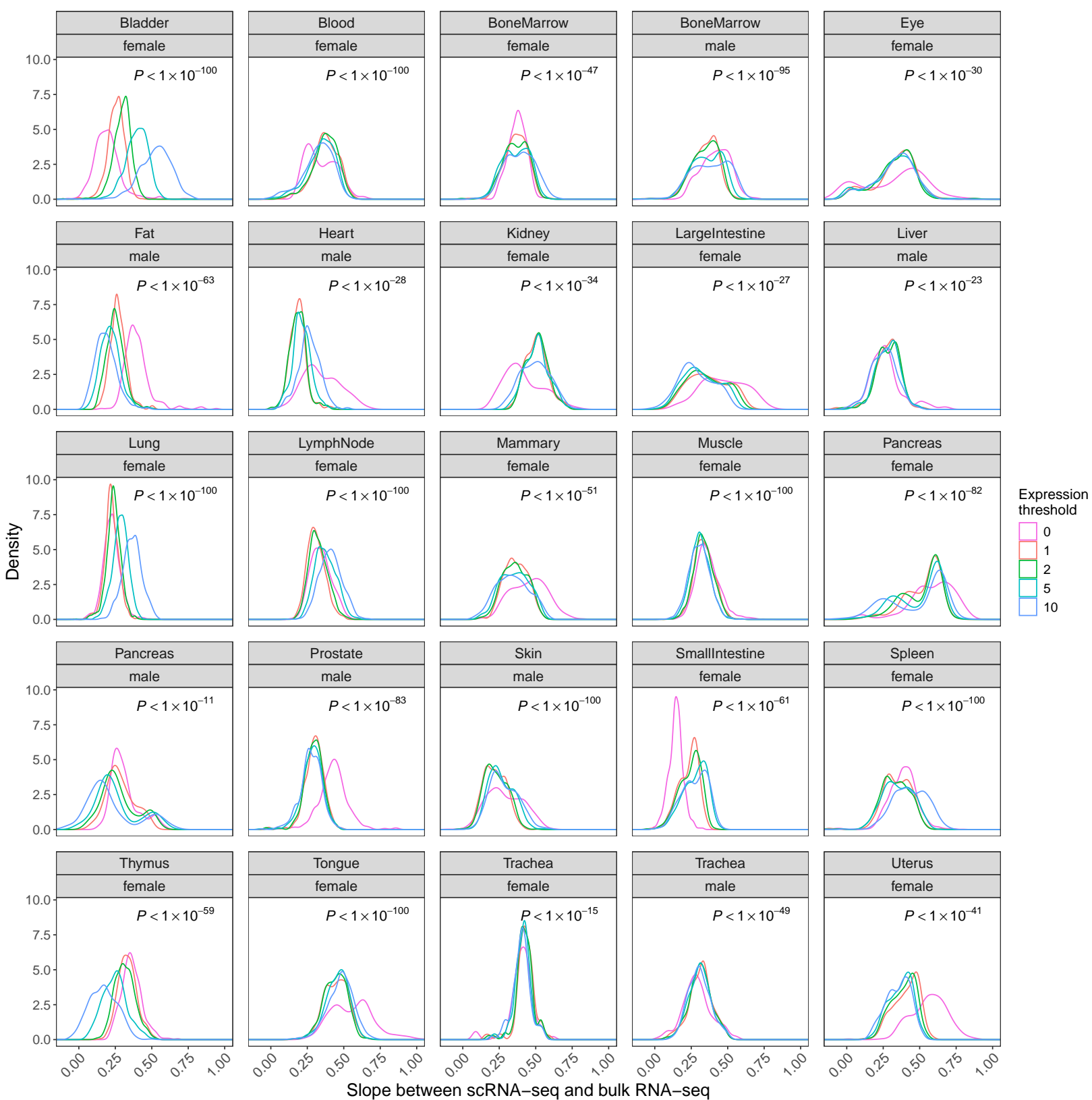

**Fig. S3**

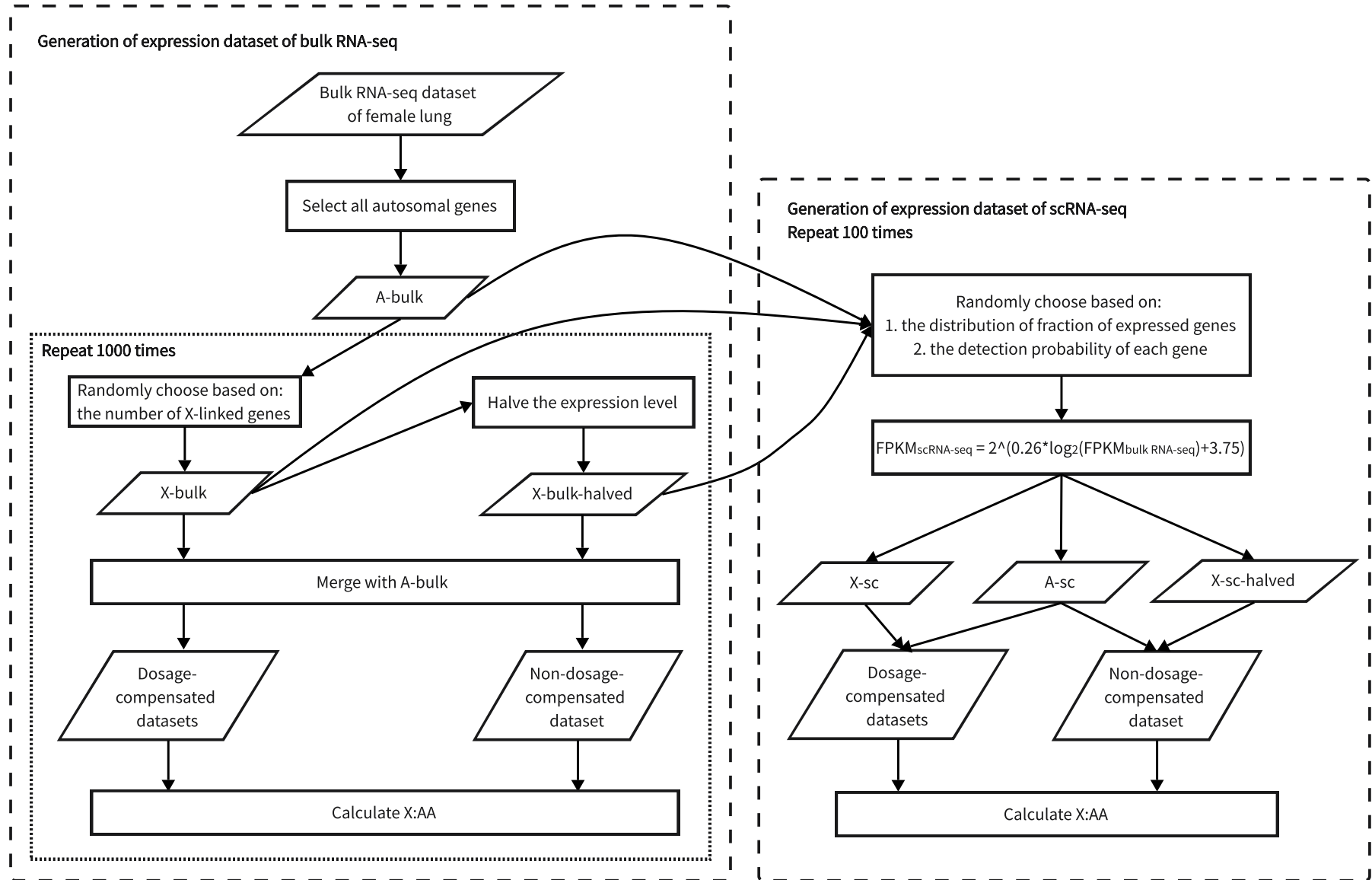

**Fig. S4**

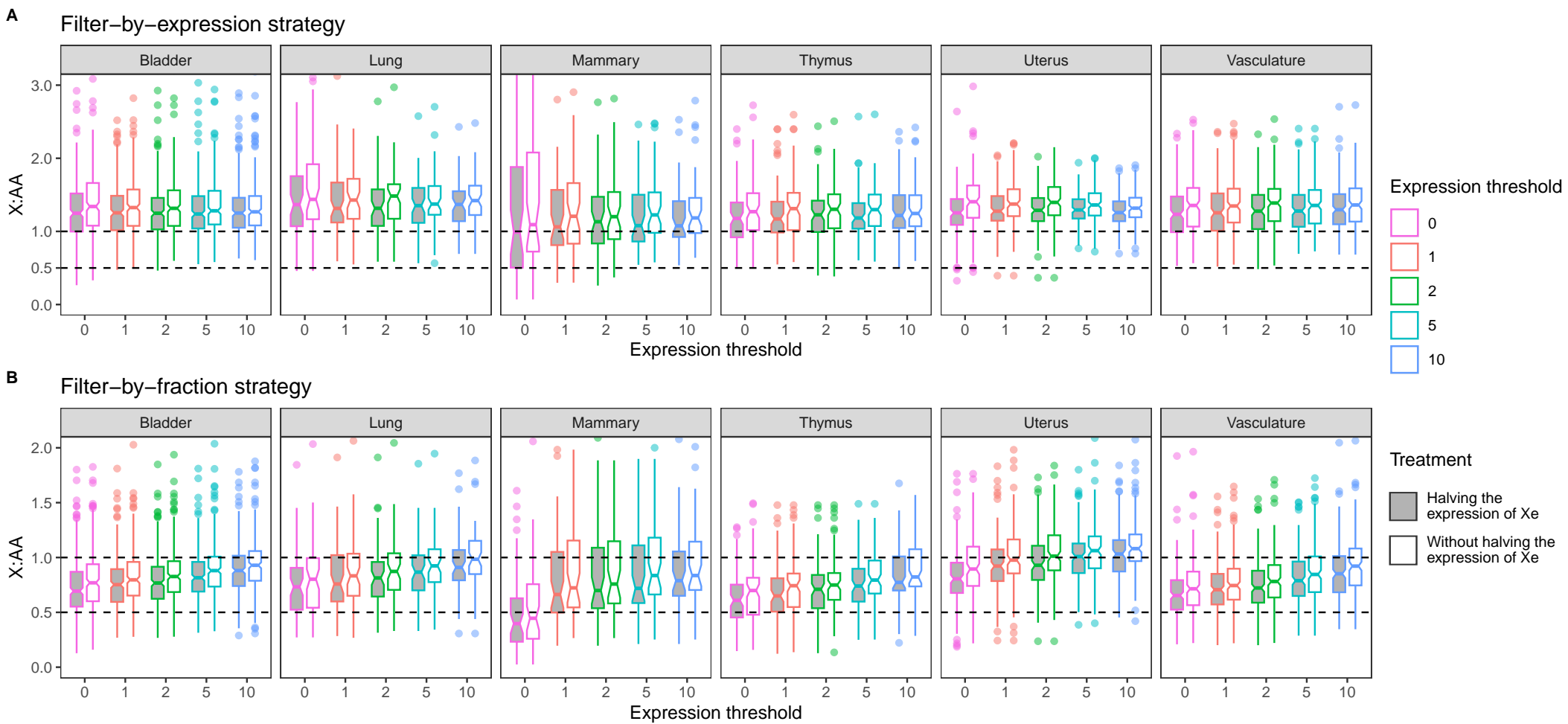

**Fig. S5**

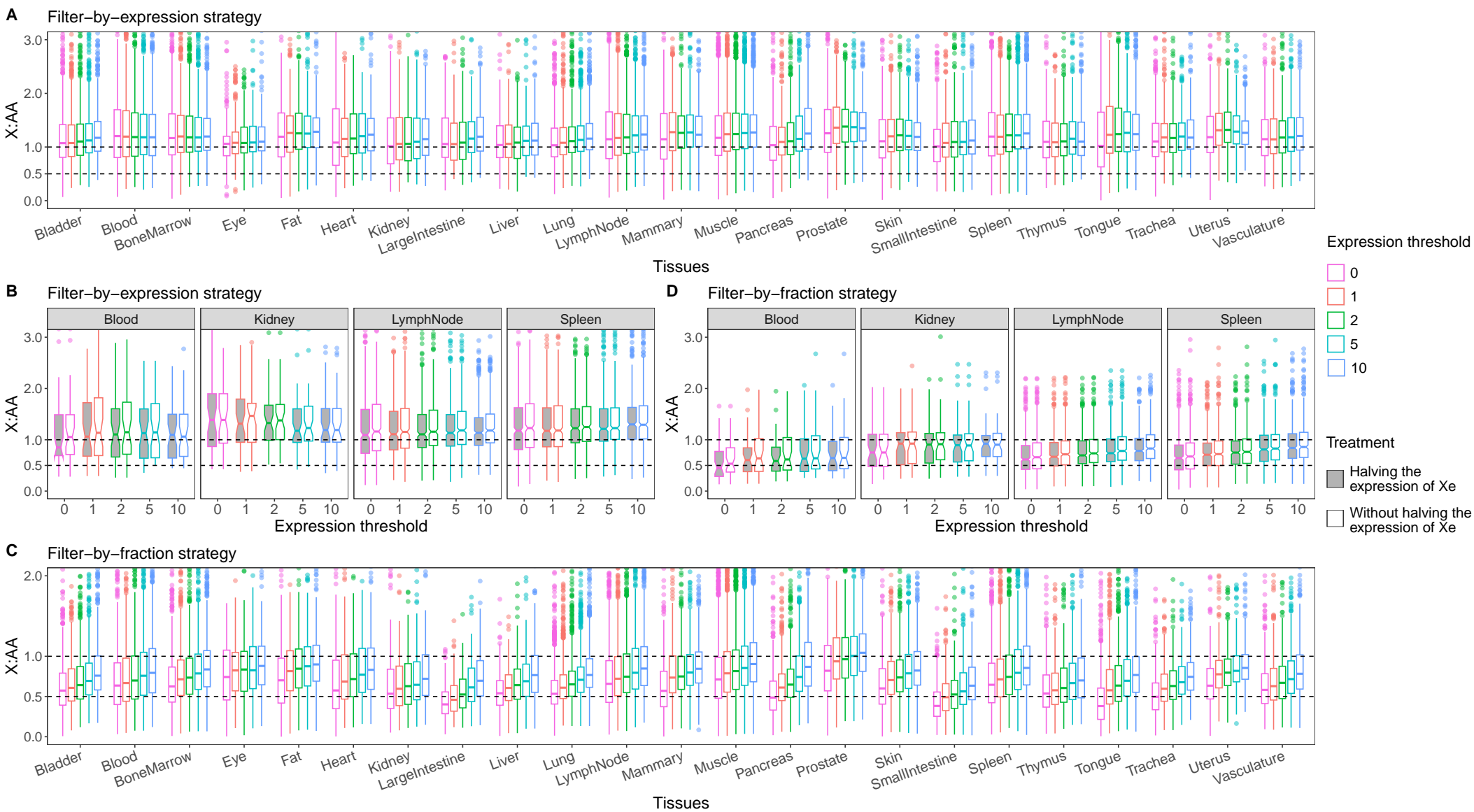

**Fig. S6**

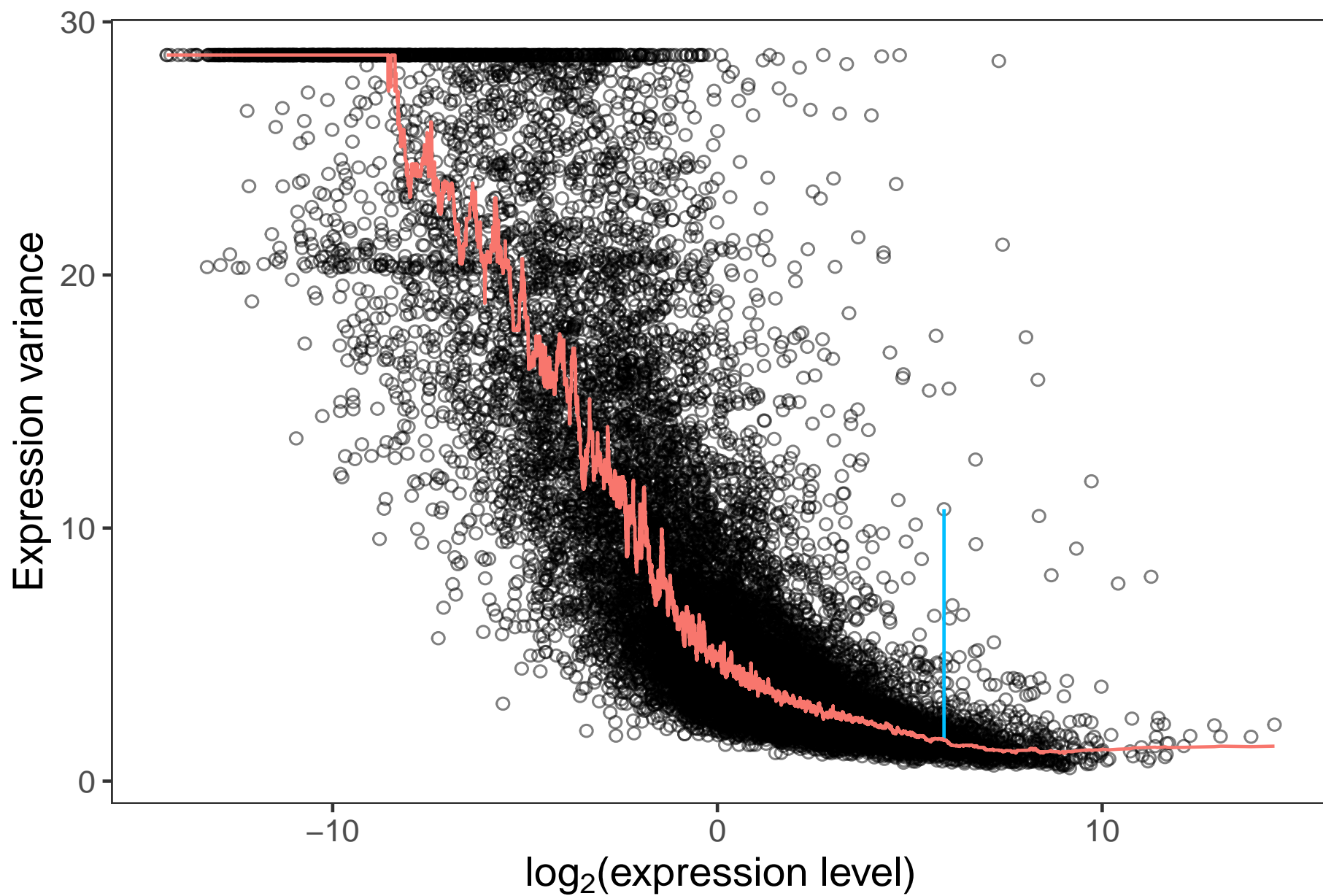

Fig. S7

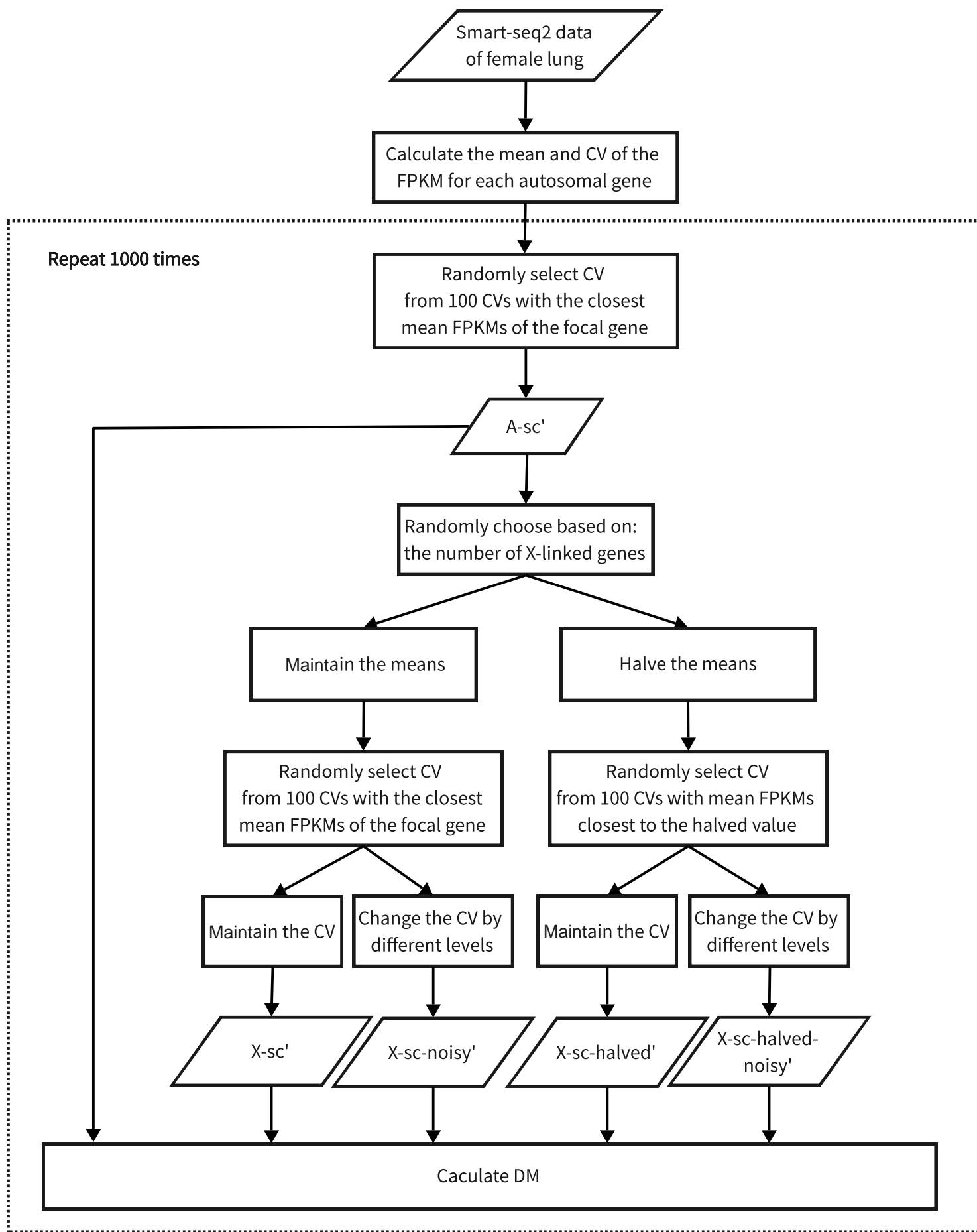

**Fig. S8**

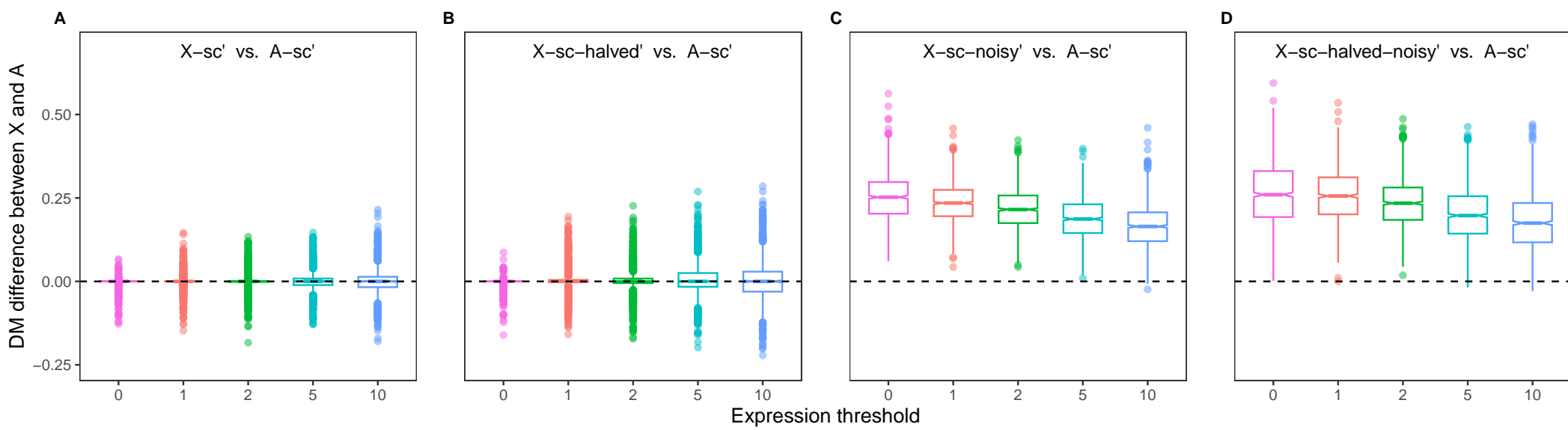

**Fig. S9**

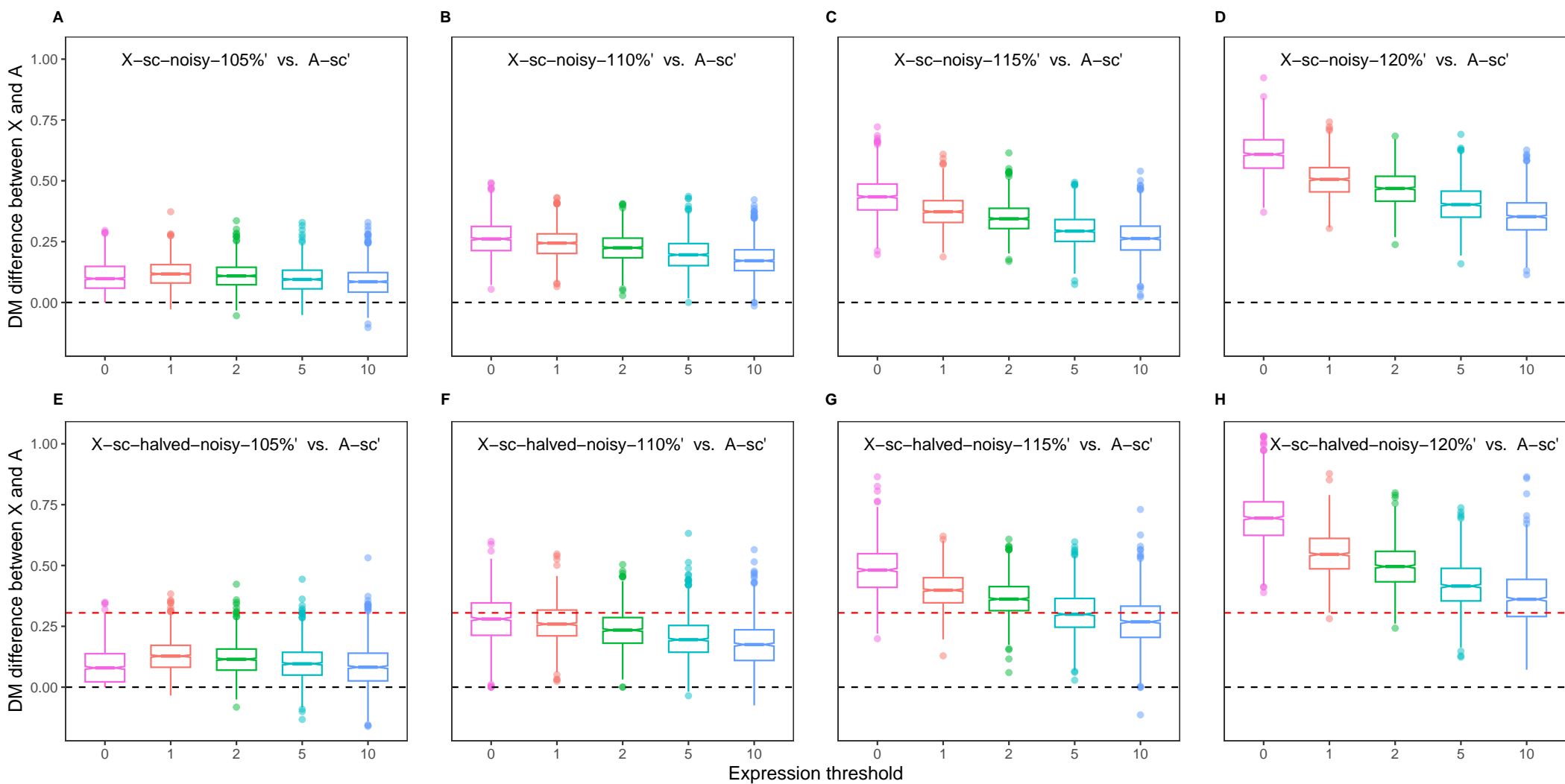

**Fig. S10**

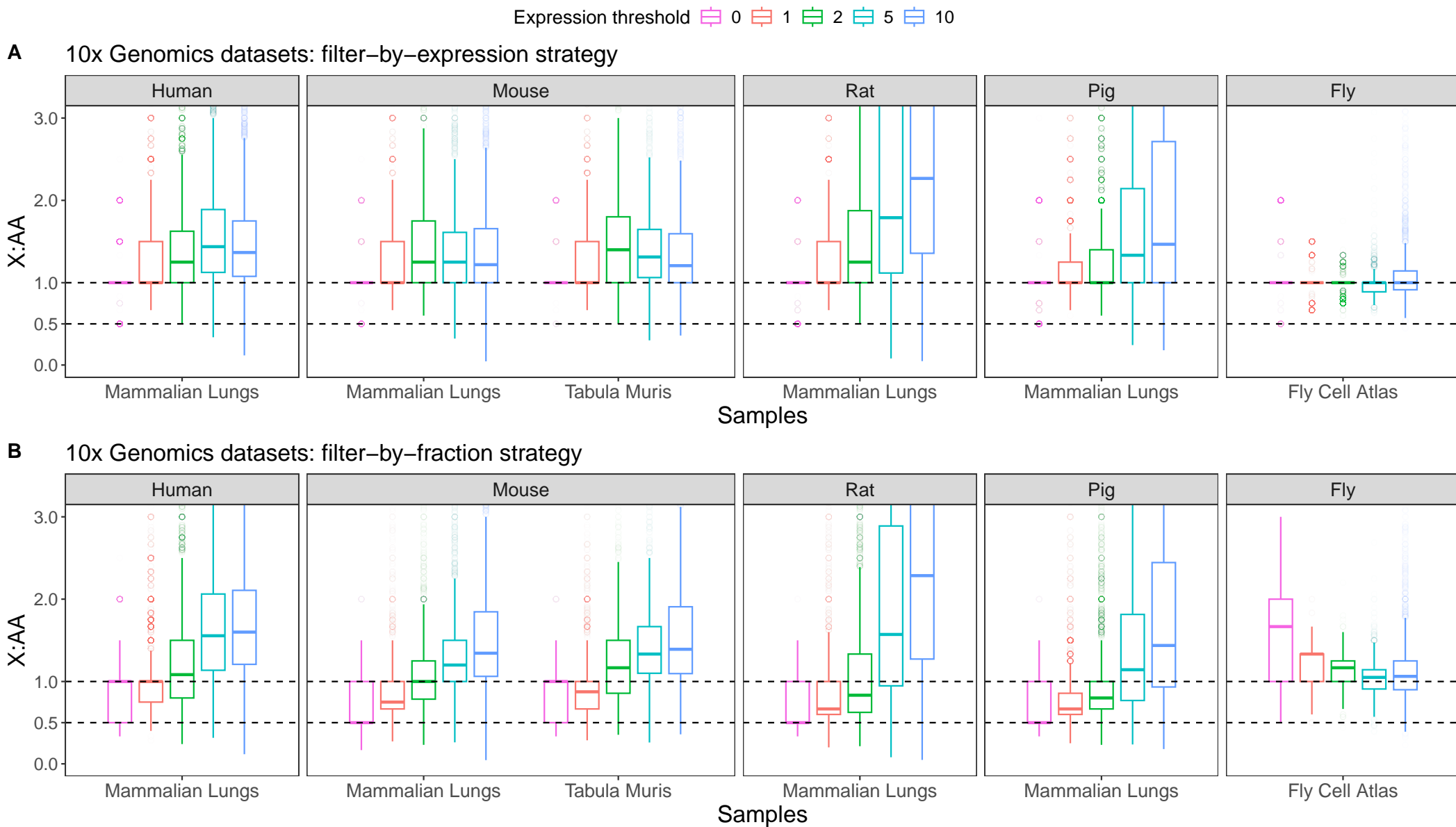

**Fig. S11**

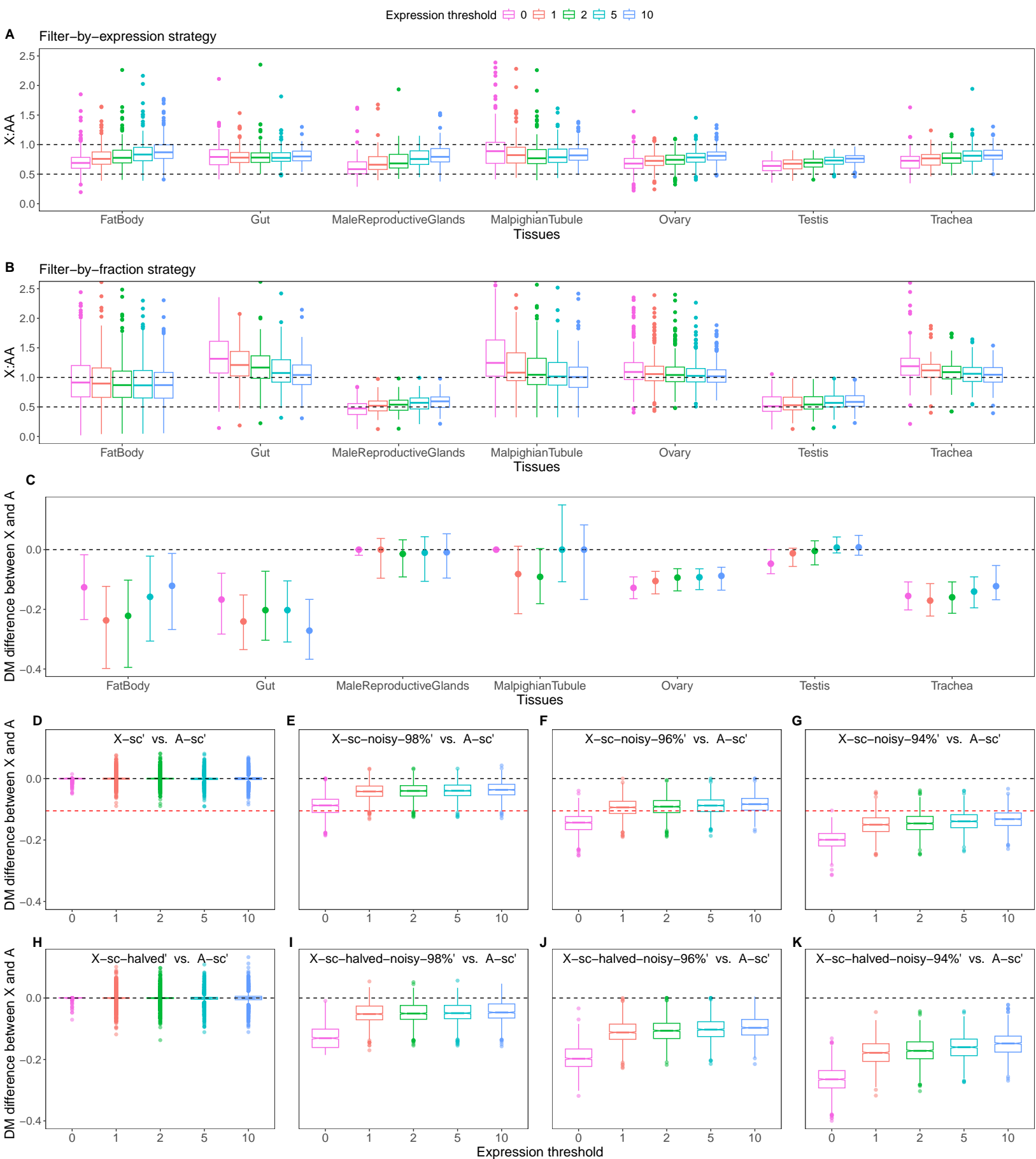

**Fig. S12**

Supplement: msaf004_Supplementary_Data [file msaf004_supplementary_data.zip › supplemental figures_v6.pdf]
